# “I Learned to Let Go of My Pain”. The Effects of Mindfulness Meditation on Adolescents with Chronic Pain: An Analysis of Participants’ Treatment Experience

**DOI:** 10.3390/children4120110

**Published:** 2017-12-15

**Authors:** Danielle Ruskin, Lauren Harris, Jennifer Stinson, Sara Ahola Kohut, Kathryn Walker, Erinn McCarthy

**Affiliations:** 1 Department of Anesthesia and Pain Medicine, The Hospital for Sick Children, Toronto, ON M5G 1X8, Canada; jennifer.stinson@sickkids.ca; 2 Child Health Evaluative Sciences, The Hospital for Sick Children, Toronto, ON M5G 1X8, Canada; lauren.harris@sickkids.ca (L.H.); erinn.mccarthy@sickkids.ca (E.M.); 3 Lawrence S. Bloomberg Faculty of Nursing, University of Toronto, Toronto, ON M5T 1P8, Canada; 4 Medical Psychiatry Alliance, The Hospital for Sick Children, Toronto, ON M5G 1X8, Canada; sara.aholakohut@sickkids.ca; 5 Department of Psychiatry, University of Toronto, Toronto, ON M5T 1R8, Canada; 6 Division of Paediatric Medicine, The Hospital for Sick Children, Toronto, ON M5G 1X8, Canada; katie.walker@sickkids.ca

**Keywords:** mindfulness, acceptance, adolescence, chronic pain, qualitative analysis

## Abstract

Chronic pain can lead to significant negative outcomes across many areas of life. Recently, mindfulness-based interventions (MBIs) have been identified as potentially effective tools for improved pain management among adolescents living with pain. This study aimed to explore the experience of adolescents who participated in an eight-week mindfulness group adapted for adolescents with chronic pain (MBI-A), and obtain their feedback and suggestions on group structure and content. A mixed method design was used employing qualitative data from focus groups and data from a satisfaction questionnaire. Focus group data were transcribed and analyzed using inductive simple descriptive content analysis. Of the total participants (*n* = 21), 90% (*n* = 19) provided feedback by completing satisfaction questionnaires and seventeen (*n* = 17) of those also participated across two focus groups. Analysis of the focus group transcripts uncovered six themes: mindfulness skills, supportive environment, group exercises (likes and dislikes), empowerment, program expectations, and logistics. Participants reported positive experiences in the MBI-A program, including support received from peers and mindfulness skills, including present moment awareness, pain acceptance, and emotion regulation. Group members suggested increasing the number of sessions and being clearer at outset regarding a focus on reduction of emotional suffering rather than physical pain.

## 1. Introduction

Pediatric chronic pain is prevalent and can lead to significant life disruption across school, social, physical, and family activities [1,2]. Quality of life can suffer and has been shown to be even lower among children with chronic pain than children diagnosed with cancer [3]. Youth with chronic pain are more likely to develop mental health disorders, such as anxiety and depression, as compared to youth without chronic pain [4,5,6], with 44% of hospital-admitted youth with a primary diagnosis of pain meeting criteria for a co-morbid psychiatric diagnosis (anxiety, depression, somatic disorder, post-traumatic stress disorder (PTSD)) [7]. Typical interventions for chronic pain focus on increasing function through the integration of pharmacological, physical, and psychological modalities [8]. Chronic pain often persists, despite our best treatment efforts [9], necessitating interventions that improve tolerance of symptoms and reduce pain-related distress. 

Recently, mindfulness-based interventions (MBIs) have emerged as a treatment option for this purpose given their versatility in targeting both physical and emotional distress. Mindfulness has been defined as the act of ”paying attention, on purpose, in the present moment, and nonjudgmentally” [10,11]. Individuals with chronic pain are taught to approach rather than avoid painful sensations, and to detach from catastrophic cognitions (“I can’t bear the pain”) and emotions (anxiety, frustration) that often accompany and exacerbate pain [12]. Other strategies, including thought diffusion, present moment awareness, and acceptance are also taught [12]. Over time, participants learn that while pain may be unavoidable, suffering and distress are optional [13].

Meta-analytic reviews of MBI’s for adults with chronic pain have reported effect sizes in the small to medium range for diminished pain intensity and for reduced mood/anxiety symptoms along with effect sizes in the medium to large range for improvements in pain acceptance [14,15]. Of the few studies evaluating MBIs for children with chronic pain, most studies have been published in the last 3 years [14,15,16]. These MBIs vary in number of sessions provided (e.g., 6–8), session duration (e.g., 60–120 min), outcomes assessed (e.g., health-related quality of life, anxiety, mood, etc.), and population (e.g., headache only [14] or mixed pain conditions including somatoform disorders) [16]. Aside from one study with high attrition rates [17], the majority of MBIs for this population have been shown to be feasible and acceptable [18]. In terms of effectiveness outcomes, results are mixed. Hesse et al. demonstrated significantly improved depression and parent reported quality of life, but no changes in pain among 20 children with recurrent headaches following completion of an eight-week MBI program [14]. A study comparing an eight-week MBI to a wait list control in adolescents with chronic pain failed to detect differences between groups on pain intensity, emotional distress, or quality of life, but found post-mindfulness session reductions in salivary cortisol [16]. Another study delivering a six-week MBI to adolescents with chronic pain did not find post session differences in pain intensity or mood, but found non-statistically significant differences in functional disability that resulted in a small effect size [19]. Finally, a recent study on the MBI-A that is the focus of the current paper found significant post-intervention improvements following an eight-week MBI in the areas of coping with stress, body awareness, and pain acceptance, but did not see post session changes in pain intensity or emotional variables [15]. Few studies assessing the impact of MBIs for adolescents with health conditions have employed formal qualitative methodologies (i.e., semi-structured focus groups or individual interviews) to provide in depth analysis of participants’ experience of the intervention. Those that have used qualitative analysis generally identified similar themes, including participants’ sense of improved well-being, self control, and enhanced interpersonal relationships [20,21,22].

The MBI-A program that is described in this paper is an eight-week mindfulness program with content specifically that is adapted for adolescents with chronic pain. The MBI-A has been shown to be feasible and acceptable to adolescents with chronic pain and had superior retention (defined by number of participants who completed treatment) when compared to other MBIs delivered to this population possibly because content was adapted to their condition [23]. From 2014–2015, the MBI-A program was piloted with a focus on feasibility outcomes with clinical outcomes as secondary objectives [15]. The program was found to be feasible to implement. Participants showed statistically significant improvements (*p* > 0.05) in pain acceptance from baseline to the three-month post-intervention follow up, and statistically significant improvements in body awareness (*p* = 0.03) and coping with stress (*p* < 0.01). Pain catastrophizing was found to be an inverse correlate of pain acceptance, however the small sample size and lack of an attention-control group limit the significance of these findings, highlighting a need for more rigorous research on clinical outcomes.

As MBIs for adolescents with chronic pain begin to be subjected to more rigorous randomized controlled trials (RCTs) [19], it is important to understand participants’ experience of the MBI and elicit their feedback, so that interventions can be further refined and improved. No studies describing mindfulness interventions for pediatric chronic pain have employed formal qualitative methodologies to document participants’ experience of the group. Therefore, the objective of this study is to describe the experiences of adolescents who participated in an MBI program for adolescents with chronic pain—the MBI-A [15] qualitatively, using a focus group methodology and by administering a satisfaction questionnaire. By eliciting the detailed experiences of participants, including the benefits and challenges of using mindfulness for pain management, this study aimed to build upon the current research and to provide new insights into the perspectives of participants. This study is unique to previous and ongoing research on the MBI-A program, as it focuses heavily on qualitative comments from participants as opposed to quantitative outcomes. Ultimately, these results will be used to refine future adaptations of the MBI-A program, and help to inform other mindfulness programs for adolescents with health conditions.

## 2. Methods

### 2.1. Study Design

A qualitative descriptive study [24] utilizing a focus group methodology [25] with adolescent participants who completed the MBI-A program was conducted to elicit detailed feedback from participants about their experience and any likes/dislikes about the group content and format. One focus group was conducted following the Fall MBI-A session (*n* = 8), and the other focus group was conducted following the Spring MBI-A session (*n* = 9). 

### 2.2. Participant Recruitment Procedure

Participants were recruited from a major pediatric tertiary care center’s multidisciplinary chronic pain clinic in Canada. Patients were deemed to be eligible if they were between the ages of 12 and 18, and were diagnosed with a chronic pain condition. Patients were excluded from participating in the study if they had a severe cognitive impairment that would impede ability to participate in the mindfulness group, based on consultation with the patient’s treating health care provider.

Ethics approval was obtained from the institution’s Research Ethics Board (No. 1000045165). Patients at this hospital’s chronic pain clinic were approached to participate immediately following their scheduled pain clinic appointment. Eligible patients were provided a letter outlining the purposes of the study and the nature of the MBI-A program. All of the patients were either met in person or contacted by phone by the Clinical Research Project Coordinator (CRPC) or the Clinical Research Project Assistant (CRPA) to share additional information about the study. Informed, written consent was obtained from all participants. 

### 2.3. Intervention

Adult-based traditional MBI programs for chronic pain, modeled after Jon Kabat Zinn’s mindfulness based stress reduction courses, typically consist of weekly two and a half-hour sessions and homework six days a week, 45 min a day [26]. This adult chronic pain MBI program was modified based on recommendations from several previous MBI studies with adolescents [22,27]. Specifically, consistent with the recommendations from Semple, Lee, and Miller [27], these modifications include reduced expectations for daily practice, shorter duration of sessions, and a greater focus on experiential, multi-sensory activities to illustrate mindfulness concepts [28]. Additionally, as recommended by Semple, Lee, and Miller, MBI concepts were presented in a developmentally tailored manner [27]. For example, while Jon Kabat-Zinn’s traditional definition for mindfulness was included in the program [11], the language presented was more accessible to adolescents such as “trust the process” and “patience”, while still including more traditional terminology such as “non-judgment” and “compassion”. Experiential activities were used to illustrate abstract mindfulness concepts. For example, the concept of “pain with resistance leads to suffering” was illustrated by passing around finger traps to participants to experience the multiple repercussions of experiential avoidance (e.g., “getting stuck”, less ability to engage in the present moment, while energy is spent struggling with experience). In contrast to one previous MBI-based study, which minimally tailored MBI program content to address headache pain [14], the MBI-A was significantly adapted to address pain (e.g., approaching versus avoiding pain in body scan/ice cube meditation and metaphors to illustrate pain acceptance). The full MBI-A curriculum, including more detail on modifications from adult content, along with detailed session content is outlined in Ruskin et al. [23].

The MBI-A group ran after school for weekly two-hour sessions over the course of eight weeks. Sessions were led by two of the authors (DR, KW) who maintain personal meditation practices. Sessions incorporated mindfulness meditations, exercises, and activities that were adapted specifically for pediatric chronic pain and focus on skill building. Topics included: mind-body connection, the effects of stress on pain, living in the present moment, focused awareness, responding versus reacting to pain and/or difficult situations, approaching and co-existing with chronic pain, non-judgment, gratitude, kindness, and compassion towards self and others.

The group also encouraged a regular daily meditation practice. In addition to the teen sessions, a parenting component was also offered in the form of a one-time parent workshop occurring separately, but simultaneously to the teen session, as a means to reinforce the adolescents’ coping strategies. Specific mindfulness skills that were introduced to parents included the concept of “responding versus reacting”, using the STOP meditation practice (Stop, Take a breath, Observe one’s experience, and Proceed with one’s response [29]) to practice responding versus reacting during interactions with their teens, along with an exercise of parents using their values to guide interactions with their teens.

Treatment fidelity was addressed through the use of a semi-structured session guide that was implemented consistently for both MBI-A group programs. Further, the same facilitators were responsible for leading both groups, limiting the variability of intervention delivery between groups.

### 2.4. Data Collection

#### 2.4.1. Satisfaction Questionnaire

The Satisfaction Questionnaire was a measure developed for this study to obtain feedback from participants regarding their experience in the group. Participants completed the questionnaire immediately following the eight-week intervention. A single item asked participants to rate how satisfied they were that the group helped them cope with pain, stress, and their sense of feeling alone. Ratings were made from 0 (“not at all satisfied”) to 10 (“the most satisfied ever”). Participants also indicated whether they would recommend the group to a friend (yes/no), and finally they were asked to write down three take-home lessons they learned from the group.

#### 2.4.2. Focus Groups

One week following their participation in the eight-week MBI-A group, adolescent participants completed an audio-recorded focus group. The timeline of one week was chosen in order to allow participants time to consider the impact of the group and any suggestions for change, and to assess feedback while the group experience was still recent. Participants received a $10 gift card in recognition of their time and effort to participate in the focus group. The focus group was led by two CRPCs who were not involved in direct treatment during the eight-week group, however were involved in recruiting and enrolling participants into the study. Both of these individuals have received training on effective qualitative interviewing techniques, and had previous experience conducting focus groups.

The focus group questions followed a semi-structured interview guide. Participants were first asked for their feedback on the mindfulness group in general, and then were asked more specific questions about different aspects of the group (e.g., skills they may have gained, possible changes they see in themselves as a result of being in the group, etc.). For the complete semi-structured interview guide, please refer to Appendix A (Table A1). Immediately following the focus group, both CRPCs documented field notes to capture non-verbal data including group dynamics, personalities present, or any other observations that should be considered in the context of the focus group data. Audiotaping and transcribing focus groups of up to ten participants precluded the ability to deduce specific characteristics of focus group participants who provided the feedback (e.g., age).

### 2.5. Data Analysis

An inductive qualitative content analysis approach was used in analyzing data [24,30]. Inductive qualitative content analysis allows for a systematic classification of the data to identify categories based on patterns. By not imposing a coding schema, inductive content analysis allowed for novel insights and understanding from the participants’ perspective, grounded in their experiences [30]. Each of the two focus group audio recordings were transcribed verbatim, and at least one of the CRPCs who led the focus group reviewed transcripts to ensure accuracy. Both of the transcripts were then imported into NVivo 10 (manufactured by QSR International; software sourced in Toronto, Ontario, Canada), a qualitative software program used to organize and assist with coding transcript data [31]. A preliminary coding scheme was developed through independent transcript review followed by a consensus meeting with all three coders (DR, LH, EM). This coding scheme was then applied to the first transcript, which was coded by LH and EM together using NVivo software. Minor changes to the coding scheme were made throughout the coding to ensure all data could be appropriately captured. After these changes were agreed upon by group consensus, the second transcript was coded independently by LH and EM using the updated coding scheme. Inter-rater reliability from the independent coding was established at 94% agreement. Disagreements were resolved via discussion between LH and EM. When the two coders were not able to resolve the disagreement, a third coder (DR) was included in the discussion. All of the discrepancies were resolved using this process. When new codes emerged, LH and EM discussed these with the lead author and previously coded transcripts were reviewed with the updated coding scheme to ensure no data were missed. Once both interviews were coded, the final codes were then reviewed by LH, EM, and DR, and all of the codes were categorized into main categories based on group consensus. All of the authors reviewed the descriptions of the categories with exemplar quotes to provide feedback, ask questions, and ensure that the categories were grounded in the data.

Descriptive data (mean, standard deviation) were provided for quantitative data from the satisfaction questionnaire. Qualitative data from satisfaction questionnaire (i.e., three take-home messages) were reviewed independently by two of the analysts (EM and LH), and final categories were developed based on group consensus (EM, LH, and DR). Finally, demographic data are presented for the 17 individuals who participated in the focus group, given that focus group data is the main target of this paper. The reader is referred to a prior publication (Ruskin et al., 2017 [15]) for demographic information on the full sample.

## 3. Results

A total of 21 participants completed the MBI-A group, in two groups. The first group ran through Fall 2014 (*N* = 10), the second group ran through Spring 2015 (*N* = 11).

### 3.1. Participants

All of the study participants were invited to provide feedback by completing the satisfaction questionnaire. Of the 21 total participants, 90% (*n* = 19) provided feedback by completing satisfaction questionnaires, and seventeen (*n* = 17) of those also participated in the focus group. See Table 1 for participant demographic information on the 17 focus group participants. Participants were unable to attend the focus group due to illness (*n* = 2), or other unspecified reasons (*n* = 2). Focus groups lasted between 60 and 65 min.

In order to determine whether differences existed between those who attended the focus group and those who did not, the attendance record and satisfaction scores were compared. Focus group non-attendees had similar average session attendance as compared to focus group attendees (6.00 vs. 6.47 sessions attended, respectively) along with similar average satisfaction scores (8.00 vs. 8.32, respectively).

### 3.2. Qualitative Analysis

**Figure 1:**
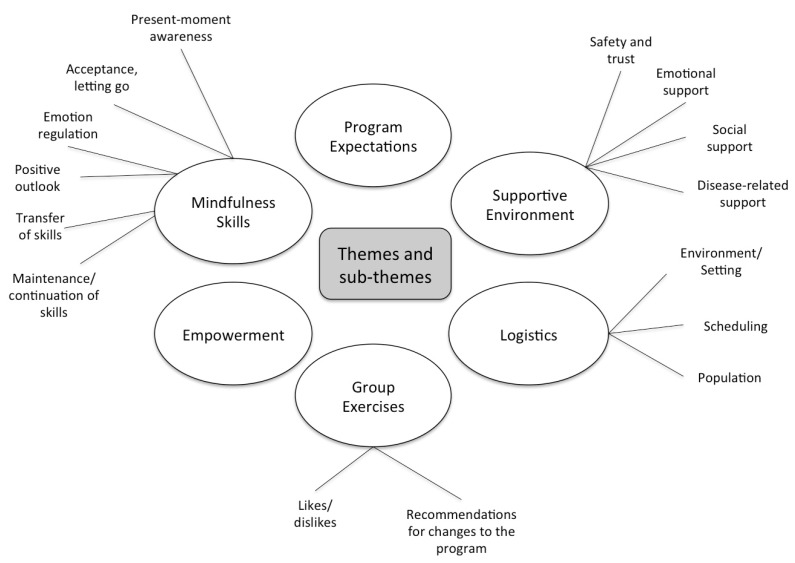
Themes and sub-themes resulting from qualitative analysis of focus groups.

Qualitative analysis of focus group transcripts revealed six main themes: Mindfulness Skills, Supportive Environment, Group Exercises, Empowerment, Program Expectations, and Logistics. Some of these themes include multiple subcategories that reflect a variety of ideas within the primary theme (Figure 1). Table A2 provides additional illustrative quotations for each theme and sub-theme.

#### 3.2.1. Mindfulness Skills

Participants described how the Mindfulness group helped them to be more present in the moment, to accept their reality and move forward despite the pain, to better regulate their emotions, and to cultivate a positive outlook. They discussed specific skills learned along with the stressful situations in which they have applied these skills to better manage distress (sub-category “transfer of skills”). Each of these sub-categories will be described below. 

##### Present-Moment Awareness

Participants described an improved ability to notice their thoughts, sensations, and emotions. As one participant stated: “*I started noticing things more like the water on my body when I showered or even little stuff like ‘oh I am feeling angry now, why am I feeling angry?’ I try to figure it out…*” (Participant, Spring group). This awareness and ability to observe one’s experience with openness rather than reactive judgment also applied to participants’ outlook towards their pain. Viewing pain more dispassionately allowed for participants to notice and to acknowledge the pain rather than expend energy resisting or distracting from it. As one adolescent described: “*You kind of focus on the little pain and you give it space and you just look at it with curiosity… you’re not getting all worked up because you are in pain*” (Participant, Spring group).

##### Acceptance, Letting Go

Participants described a shift in how they perceived their pain before the group when compared to afterwards. They described previously feeling controlled by the pain, whereas now they have learned to not “push away” the pain, but instead to acknowledge that the pain exists. As one participant (Spring group) stated: “*it changes… how you look at the pain all together. Before we came here, we kinda looked at [the pain] as a burden and something that we have to lug along. But now we kind of look at it like a companion…like another organ or something like that and you have to deal with it*”. As another participant (Fall group) described: “*I learned to let go of my pain…like you know what, maybe I will just forget about it and do what I want to do and now I somewhat do that instead of just sitting on the couch and just sit in my sorrow*”.

##### Emotion Regulation

Participants described the MBI-A program as something that helped with the emotional aspects of pain, rather than the physical pain sensations. They described carrying a heavy emotional burden in addition to the pain, for example frequently worrying about the pain, and feeling emotionally vulnerable in response to minor setbacks. Participants described noticing that they felt less anxious and less angry about “*every little thing*”. They described making a conscious effort to use their logical versus their emotional mind to deal with stress: “*Instead of getting all stressed and upset about what I’m stressed about…I can kinda think my way through it instead*” (Participant, Fall group).

##### Positive Outlook

Participants commented that they left the MBI-A program with a positive outlook. Patients described feeling happier after the group, which in some cases was attributed to spending time with people who can relate to one’s experiences, and in other cases, was described as a more conscious effort to focus one’s mind on positive aspects of their life. As one participant (Spring group) stated: “*I noticed I am a lot happier. Before, I was feeling really down because I wanted to do bike rides and stuff that you can’t really do. Coming here it was like there are other people who you can complain to and it’s like ‘oh, you feel the same way!’ It kind of makes you feel better in a strange way*”. Participants also described that this positive outlook lasted beyond the time of the group. “*It was just in general being more positive about a lot of things whether it’s like family issues or anxiety or pain or anything along those lines, it was just being more positive, more understanding about a lot of things and not letting every little thing get to you and make you anxious or angry or depressed*” (Participant, Spring group).

##### Transfer of Skills

Participants provided examples of when mindfulness skills helped with other aspects of their lives, in some cases unrelated to pain. For example, one participant used mindfulness strategies in their part-time job in customer service when dealing with a frustrated customer. Another participant used meditation to help fall asleep: “*I knew that if I ever had a hard time falling asleep cause of pain, I could just do meditation and it would help relax me*” (Participant, Spring group); and mindful breathing strategies to support emotion regulation in a stressful classroom situation: “*I get annoyed by loud noises so when I am in class I’m just like take a deep breath and I can filter it out better*” (Participant, Spring group).

##### Maintenance or Continuation of Skills

When asked about ways to maintain mindfulness in their lives after the group ends, many participants described that their way of thinking had adapted so that mindfulness had become part of who they are. Mindfulness was not perceived as something that requires dedicated time; rather, participants viewed it as a way of thinking and feeling. As one participant stated: “*It’s kinda just become a part of me now*” (Participant, Fall group), “*Yeah, it’s become natural… It’s almost like engraved up there*” (Participant, Fall group).

#### 3.2.2. Supportive Environment

Participants described the group as a supportive environment where they felt safe to openly express themselves and share feelings with one another. Participants discussed three different types of support received (emotional, social, disease-related), and how it helped them. Group members also emphasized the importance of meeting others who have chronic pain and the safe and trusting group environment that helped them to openly express themselves.

##### Safety and Trust

Participants described how the group fostered a sense of trust and openness with each other. “*It was just like everyone was really open with each other and it wasn’t like you had to hold anything back, like you could talk or, if you did say anything, you could say anything*” (Participant, Fall group). Group members stated they treated each other respectfully and were sensitive and considerate of everyone’s privacy. One adolescent reported they felt their “story” was believed and that group members did not doubt them or the validity of the information they shared. Overall, the participants reported they were able to share freely with one another without any fear of judgment. 

##### Emotional Support

Participants felt supported by group members, which facilitated their ability to connect with one another. Group members commented that they were able to give and receive feedback to each other and that everyone could relate in some way. “*It was good to get, like feedback ‘cause everyone is really like good to each other with like giving feedback*” (Participant, Fall group). When asked what they enjoyed most about the mindfulness group, one adolescent replied: “*Being able to talk about it, how we feel about our pain, about things that bothered us*” (Participant, Fall group).

##### Social Support

The majority of participants shared that “*they felt less alone*” as a result of participating in the mindfulness group. Adolescents reported they now had new friends they could reach out to for support both within and outside of group sessions. “*It just seems like everyone here became like best friends and like you can say anything*” (Participant, Fall group). Weekly sessions provided a forum for members to share their stories and seek feedback from each other without feeling like they were a burden. One adolescent described: “*You knew like after a long hard week, or a good one, you knew you could come back and like share your stories or something, like you had somewhere to go to tell people about it*” (Participant, Fall group). When discussing the group coming to an end, one participant spoke about the connection that they all shared and how it felt like they had to leave their friends.

##### Disease-Related Support

Participants spoke about the benefits of participating in a group with others who also have chronic pain. Taking part in the mindfulness group exposed them to different perceptions and enabled them to learn new strategies to manage their chronic pain from others that are in similar circumstances. The adolescents reported a sense of connection with one another, that they were all united by a common bond—their pain. One member shared that prior to the group, they had not met anyone who had chronic pain. Another adolescent shared they now felt an increased sense of support knowing there were others who also live with similar disease-related restrictions. “*I feel like other people—they don’t really know what you are going through, you have to like consistently explain it to them. Like in this group, everyone can agree with what you’re saying*” (Participant, Spring group). Moving forward, one participant suggested that whenever members feel discouraged, to recall this group and know that they are not alone and there are others like them who understand. “*’Cause you remember the people that were there, so you’re like…whenever you are like oh this sucks and no one understands, you can be like wait a second, I don’t remember their names anymore but they understood*” (Participant, Spring group).

#### 3.2.3. Group Exercises

Group members discussed what they liked about the group (variety of exercises, experiential component, and identified specific “favorite” exercises). It was evident that some group members had mixed opinions about exercises, suggesting that the same exercise could be perceived differently by different participants. Recommendations for changes to the program were also identified.

##### Likes and Dislikes

Participants enjoyed the different methods used to illustrate mindfulness concepts (including experiential activities, metaphors, art, music, and drama): “*they had very, very good methods of getting across the kinds of lessons we were learning*” (Participant, Spring group). Participants also enjoyed the variety of exercises: “*every single week it was just always something new that was fun*” (Participant, Spring group). Participants enjoyed most activities provided; however, some favorites included the “weather report” (describing weather patterns as a method of becoming aware of internal states), practiced at the beginning of every session “*I find that helpful ‘cause it is really hard to describe how you are feeling some times and then when you put it into weather, I don’t know, it is easier*” (Participant, Spring group). The song played at the beginning of each session (with a volunteer to play their song each week) was also enjoyed: “*it was a good way to get across who we were as a person and just the lyrics themselves—the songs were very, very helpful*” (Participant, Spring group). Participants liked meditations with a relaxing component: “*I personally like the ones …where you just kinda relax and you take in everything around you, those ones were really nice—it kinda took away the stress like you kind of let out your tension*” (Participant, Spring group). Participants generally liked the mix of formal and informal meditation. 

There were mixed responses to meditations with a greater focus on pain—for example, one participant said “*focusing on the pain, it was really cool ‘cause you got to visualize it…we drew out like how that looked and then we went back into the meditation type thing and we did everything we could to try to soothe it, instead of pushing it away or trying to make it feel different, we just tried to make it feel a little better…it was a really, really good and very enlightening exercise*” (Participant, Spring group)*.* Others disagreed “*I found those ones irritating because it brings to mind everything, I know it is mindfulness so you are supposed to take it all in, but I liked it better when we were taking it in, in steps whereas when we were doing meditations it was all at once and that was not very comfortable*” (Participant, Spring group).

##### Recommendations for Changes to the Program

Participants had helpful suggestions for future groups. They felt that it was important at some point to tell their “pain story”: “*so it is not really awkward initially, so people know who you are*”, “*I was very curious*” (Participants, Spring group). Participants suggested that this disclosure should occur voluntarily and mid-way through the sessions “*not necessarily in the first session…the first session is like dipping your toes, testing the water*”, “*but halfway when you are starting to get to know people in the group…it would be nice*” (Participant, Spring group). The second focus group proposed the idea of sending a written message that is positive and supportive to future group members from former group members “*just to help them realize it’s ok*” (Participant, Spring group). Participants also wanted a follow-up to the weather report, so that if someone’s weather was a “*torrential downpour*” (Participant, Fall group) they could have the opportunity to say why they were feeling that way, so the group could have context and understand how they might be able to help.

#### 3.2.4. Empowerment

Participants highlighted a sense of having new ways of responding to their pain, including a willingness and openness to move forward with areas of their life despite discomfort. They highlighted that while strategies do not fully resolve their pain, they felt empowered in being able to take action to help manage their pain. One participant (Spring group) described: “*It doesn’t necessarily always work* [to reduce the pain], *but at least the idea that you have done something to stop it, at least that provides a little satisfaction because you tried to do something, you are not just sitting there*”. Another participant described feeling empowered to try activities that they previously would have avoided due to a fear of causing more pain: “[The group] *kind of helped me push myself in a way. I still have like a fear that if I do something then I am going to hurt my leg again or it will just turn worse. But then I found after the mindfulness group, I am more open to trying things and I am not scared, I am open to it like ‘you know what, ok yeah I can try it, it is okay if I am a little sore afterwards*” (Participant, Spring group).

#### 3.2.5. Program Expectations

Group members described preconceptions about mindfulness, but also how their expectations regarding the hospital experience changed during their participation in the group. Some described that prior to the group, they thought mindfulness would be “*stupid*”, that they would likely “*skip sessions*” that it would be “*all about closing eyes and meditating*” and that group members would be “*crazy people*” (Participants, Spring group). However, they described that having an attitude of openness, patience and practice, allowed them to become more accepting of mindfulness so that they came to appreciate the approaches taught. A participant did comment, however, that setting realistic expectations at the outset of the group was important so that participants are not overly hopeful that mindfulness will take away pain. 

A general theme was evidenced of finding the mindfulness group different from other hospital encounters. Participants spoke about the dread of coming to hospital appointments and fearfulness that health care providers (HCPs) would not be able to help them or would disappoint in some way. However, mindfulness group was described as a place where they did not have expectations about being disappointed and generally looked forward to the group as “*having a lot of fun*”, “*learning something new and not expecting anything*” (Participants, Spring group), while being with supportive peers.

Some participants described an expectation that mindfulness was going to improve their physical pain. While some experienced improvement in their overall pain, others felt it was important for facilitators to be clear that strategies are geared towards improving the emotional distress associated with pain but not the pain itself.

#### 3.2.6. Logistics

Participants generally said the current eight-week, two-hour session MBI-A was acceptable and feasible but had specific suggestions regarding time of day that the group is offered, number of sessions, and delivery model as described below.

##### Environment/Setting

Participants enjoyed the mixture of sitting/quiet activities and active/movement activities within each session (“*I don’t like sitting in chairs too long—we got up and did stuff*”) (Participant, Spring group). However, they felt the group room was typical of a hospital room and needed more colour/paintings and brightness. Participants enjoyed posters that were on the wall during every group with key words that embodied MBI-A concepts (e.g., openness, kindness, acceptance, no judgment, patience) “*I don’t know—we all knew them—but having them there felt so good*” (Participant, Fall group).

##### Scheduling

The 4.30 pm weekday time was thought to be feasible with some participants advising for even later (5 pm) to avoid missing school and allowing for travel time. Different models of group scheduling were proposed including shortening the group to 1 h per week (vs. current 1.5 h) and instead providing two sessions (vs. current 1 session per week) “*having it once a week, you kind of start losing focus throughout the rest of the week plus having it spread out a little bit more (i.e., twice a week) gives you more time to reflect on what you learned throughout the hour*” (Participant, Fall group). For a twice weekly offering, one participant even suggested doing one of the two sessions as an online option “*maybe do one on Skype … because I would not like to miss that much school and I feel bad asking my parents to do all that*” (Participant, Spring group). Overwhelmingly, participants wanted group sessions to extend beyond the eight provided. They expressed sadness that the group was ending “*next Thursday, I am going to feel really empty*”, “*I feel so comforted*”, “*I look forward to it*”, “*It’s like a routine now.*” (Participants, Fall group). One participant (Spring group) articulated “*there was so little time between like adjusting between like there is new people around me to being like oh these people are actually really good friends of mine now … now that we are at the end of the 2 months I just don’t want to say goodbye*”. Different models were suggested including extending the group to 10 sessions, a drop-in style group that runs regularly, and provision of a yearly booster. Participants unanimously felt that the provision of an intensive week-long mindfulness course/camp would not be as effective as spacing sessions out “*I think space—because it gives you like time to kind of reflect on what you did*” (Participant, Fall group). Another participant added “*like over a span of time you actually like it seems like you’re with the people longer almost and like you see them for like 2 months opposed to like 1 week and then you forget people*” (Participant, Fall group).

##### Population

Participants appreciated being with others who have chronic pain conditions. “*Knowing that there is someone else who goes through* [pain] *on a daily basis and it is not just you, and understanding that you are not alone. That is honestly a really, really big help*” (Participant, Spring group). Participants liked having a smaller sized group (*n* = 10) “*when I first got here I saw that it wasn’t very many people, that made me feel I don’t know… better because there were less people*” (Participant, Spring group). While ages within the groups varied, a younger participant (age 13) from a group that had mainly older teens stated that she would have preferred having more participants in her group around her age “*in my personal opinion, I had no problem talking to all you guys but there should be a shorter age range… because there is a real difference*” (Participant, Fall group). Participants also were disappointed there were not more males in the group (all group members were female except one) because “*it would have added a new perspective*” (Participant, Spring group).

### 3.3. Satisfaction Questionnaires

The average rating on the satisfaction questionnaire was 8.29 ± 1.24 indicating participants felt satisfied that the group helped them to cope with their pain, stress, and feeling less alone (where 0 = not at all satisfied, and 10 = the most satisfied ever). Several themes emerged from the “take-home lessons” that participants identified on the questionnaire and are represented in Table 2 along with some exemplary quotes. These themes including social support, mindfulness skills, and pain acceptance showed overlap with themes identified in focus group transcripts. All of the participants who completed the satisfaction questionnaire (*n* = 19) indicated that they would recommend the group to a friend.

## 4. Discussion

Qualitative data describing experiences of adolescents who completed the MBI-A revealed that mindfulness skills and the group modality emerged as key elements of the MBI-A group that participants perceived to be beneficial. Given the qualitative methodology that was employed in this study, these findings do not confirm treatment efficacy, but rather highlight aspects of the intervention that participants found satisfying. Feedback from group participants provided rich information on their experiences, wants and suggestions for improvement that can inform the work of those implementing mindfulness groups for this population. Six main themes from focus groups were highlighted including: Mindfulness Skills, Supportive Environment, Group Exercises, Empowerment, Program Expectations, and Logistics. Participants’ ratings on a satisfaction questionnaire indicated that they were satisfied with the group, would all recommend the group to a friend and themes derived from qualitative comments on this questionnaire were very similar to those articulated during focus groups. Overall, this study supports and builds upon previous research demonstrating the benefits of mindfulness for adolescents with chronic pain and provides recommendations for further refinement of the program [14,16,23].

As consistent with the practices taught in mindfulness, participants described gaining skills in pain acceptance, emotion regulation, and present moment awareness. Skills were not only described as improving pain coping, but also generalized to coping with other difficult experiences, such as intense emotions, an impact that is well described in research on MBIs [12]. These findings are consistent with adult literature, which has found MBI programs to be effective in improving depression symptoms and quality of life [32]. Perhaps equally, if not more valued, was the social support provided by offering the mindfulness skills in a group context. Not only did participants feel “less alone”, they also valued learning from peers about implementation of skills (versus being prescribed skills by HCPs. The increased credibility that was derived from group members sharing their use of mindfulness skills with each other and the social support from a group format argues for implementation of mindfulness approaches in a group or peer to peer modality (versus individually with an HCP), though one study showed individual and group modalities to have equal impact on depression, anxiety, and positive well-being [33]. Group formats may be of particular benefit within the adolescent population, when individuals separate from parents and adults in favour of peers [34]. In addition, offering the MBI-A specifically to adolescents with chronic pain and tailoring content for chronic pain was viewed by participants as a benefit. This was in line with feedback from previous MBI-A pilot groups who preferred to be with teens with the same chronic condition (see Ruskin et al. 2015) [23]. This type of pain-related social support has been shown to be a predictor of reduced pain and disability within the adult literature [35]. 

Despite these benefits, several important suggestions were gleaned from focus groups. First, the participants advocated for setting clear expectations at the outset, that the MBI-A may assist with pain-related distress but may not change pain itself. While efforts were made to set this expectation early in the group, provision of written material explaining what to expect and what not to expect with mindfulness may strengthen this message. Because it may understandably take time and practice for participants to understand that MBI approaches are more about changing one’s response to experience rather than the experience itself. It is also possible that heeding participants recommendation of extra sessions (e.g., extending to 10 sessions) will permit more time for participants to engage in practice and understand this distinction.

Next, participants advocated for a more paced approach for meditations regarding “being with pain”, such as approaching areas of discomfort with an attitude of openness and softening our response to it. Given that this meditation can be challenging, modification could include: (a) starting practice with an area that is not the most bothersome (b) not needing to go “all the way in” (c) staying with the breath if things get difficult, and (d) recognizing that this exercise can take practice so that much like going to the gym—one starts slowly and builds up. Participants were also curious about other members’ stories/backgrounds and wanted opportunities, albeit voluntary, to share more about their condition or well-being. Up to this point, the MBI-A program has run without asking participants to directly share information about their condition. In our experience, this information often arises naturally, but not in detail during the course of the eight weeks. Nevertheless, an interesting suggestion from one group member was that participants voluntarily write their “pain story” on a cue card (anonymously or with their name), and that this be posted at the end of the third session for group members to read. Rather than becoming a component to be discussed formally in the group, participants suggested that they could connect on this socially outside of the group. An advantage of this approach is that it may strengthen the sense of universality among participants while using in-session time for mindfulness practice and processing of skills. In addition to benefitting from learning about peers’ stories, the act of telling one’s own story may confer its own therapeutic benefit, as described in the field of narrative medicine [36,37]. Mindfulness practices, such as that of cognitive defusion [38] (noticing the story rather than getting caught up in it) can also be employed to assist participants in decreasing the importance of the story itself, and increasing flexibility in whether or not they wish to identify with it.

While participants clearly derived benefit from the social support of the group and wanted more opportunities to share with one another, based on years of facilitating the mindfulness group it is suggested that a balance be created so that in-session time is primarily focused on teaching mindfulness concepts, active experiential exercises, and mindfulness practice, during which social connection naturally occurs. Consideration can also be given to extending the group session time and reserving the first 15 min for “snacks and social time” so that group members have opportunities to connect and deepen their relationships. A final suggestion that was unanimous across both groups was to increase the amount of sessions so that participants have more opportunities to stay connected and continue their mindfulness practice together.

### 4.1. Limitations

Several limitations should be considered when interpreting study findings. First, feedback was primarily obtained using a focus group modality rather than individual interviews. While focus groups are known to elicit greater detail and further elaboration of ideas, individual interviews can generate unique ideas and encourage disclosure (particularly around sensitive issues) [39]. A disadvantage of obtaining feedback from participants in group format is the potential for “group think” (i.e., loss of individual opinions), especially for more timid individuals, as participants tend to regress to the mean. Therefore, we may not have maximized the opportunity to obtain feedback that participants may have been more comfortable expressing in a one to one context. Nevertheless, participants appeared willing to disagree with one another during focus group sessions as evidenced by the range of opinions expressed regarding group exercises (group members were willing to say they liked exercises that others disliked). Additionally, given that focus groups were audiotaped, we were unable to identify specific individuals, and thus could not provide characteristics of who provided comments (e.g., age or individual participant), which may have been of interest to the reader. Furthermore, because not all participants attended the focus groups, we did not obtain feedback from all study participants. It is possible that non-attendees represented participants who were least engaged in the group (and thus whose feedback would have been especially important to obtain). However, given the similar attendance rates and satisfaction scores between attendees and non-attendees, it is less likely that focus group non-attendees lacked engagement in the group. Finally, although efforts were made to reduce demand characteristics associated with participants completing the satisfaction questionnaire in the room (e.g., ID numbers were used, facilitators were preoccupied with tidying the room while participants completed the questionnaire) participants may have felt pressured to respond positively on this measure. Future studies may wish to administer satisfaction questionnaires in a more anonymized manner (e.g., participants complete the questionnaire online with data sent back to the research coordinator).

### 4.2. Future Directions

Given that attendees clearly enjoyed and benefited from the social support of the group, future study designs should assess social support as an active control condition to determine whether mindfulness approaches within the MBI-A confer benefit beyond social support. In addition, modalities that are more accessible, such as online platforms, can potentially reduce some of the barriers that participants identified (e.g., missing school to attend the group, burden on parents to bring them to the group). Furthermore, given participants’ feedback that group sessions should be extended beyond eight, providers should consider offering more sessions that may be shorter in duration (e.g., from 1.5 h vs. 2 h). Finally, investigating the potential of booster sessions or drop-in formats following completion of the initial MBI-A is warranted given feedback that participants desired these options.

## 5. Conclusions

This study explored the experiences of participants in a MBI-A program for adolescents with chronic pain. The mixed methodology design revealed qualitative themes that were relating to Mindfulness Skills, the Supportive Environment, Group Exercises (likes and dislikes), Empowerment, Program Expectations, and Logistics. The group was found to be a feasible intervention for adolescents with chronic pain, and the participants perceived value in the social support that they felt from their peers. The recommendations made by participants in this group will be valuable for healthcare providers working with adolescents, particularly those who aim to develop or adapt their own MBI program.

## Figures and Tables

**Table 1:** Demographic characteristics of the adolescent sample attending the focus group (*n* = 17).

Characteristic	Results
Age, years, mean ± standard deviation (SD)	15.8 ± 1.2
Gender, *n* (%)	
Female	16 (94%)
Male	1 (6%)
Types of chronic pain, *n* (%)	
Musculoskeletal	10 (59%)
Complex regional pain	
Syndrome	2 (12%)
Abdominal	1 (6%)
Headache	1 (6%)
Pelvic	1 (6%)
Mixed ^1^	2 (12%)
Duration of pain ^2^, months, mean ± SD	33 ± 21

^1^ Mixed pain includes features of neuropathic and musculoskeletal pain; ^2^ Duration of pain data available for 16/17 participants, data missing for one patient.

**Table 2:** Major themes from the satisfaction questionnaire.

Theme	Subthemes	Adolescents (*N* = 19)	Exemplar Quotes
Social support	Feeling less alone Sense of feeling understood by peers Sense of shared experience	12 (63%)	*“Throughout this year I’ve been dealing with chronic pain. I’ve felt more alone than I ever have before. I thought nobody on Earth had what I have but I learned that I am not the only one” (Participant, Spring group, age 15)*
Mindfulness Skills	Increased awareness Meditation skills Emotional awareness	12 (63%)	*“I am going to use the meditation strategies that we used to help calm me down and try to ease my pain. I am going to spend more time being mindful when I am doing things like eating, showering or walking” (Participant, Fall group, age 15)*
Shift in Mindset	Using their “wise mind” Thinking with a balanced mind (logical, emotional) Increased positive outlook	9 (47%)	*“Sometimes our minds intensify the pain but by taking control, those same minds can decrease it” (Participant, Spring group, age 15)*
Skills to Cope with Pain	Strategies to cope with pain Knowledge that there are ways to manage the pain	6 (32%)	*“How to cope when my pain is uncounted” (Participant, Spring group, age 16)*
Pain Acceptance	Letting go of their pain How to accept pain	6 (32%)	*“I am trying to understand that pain is a part of me now but it doesn’t make me or control me” (Participant, Fall group, age 17)*

## Appendix A

**Table A1:** Focus group semi-structured interview guide.

Broad Question	Probing Follow-Up Question(s)
1. How did you feel the mindfulness group went?	What did you enjoy about it? What didn’t you enjoy about it?
2. What skills do you think you gained from being in the mindfulness group?	Are you able to handle pain or stress differently than before you did the group? Did you use these skills for pain or other things in your life? (stress, anxiety, anger etc.)
3. After doing the group, what does mindfulness mean to you?	N/A
4. What did you like best about the program?	What were some of the good things that came out of the group? Were there some group activities/exercises that you really liked? Why?
5. What did you like least about the program?	Were there any aspects of the group that you disliked or could be improved? Were there some group activities/exercises you really disliked? Why?
6. What changes did you see in yourself as a result of being in the group?	Your response to stress? Pain? Upsetting situations? Being with other group members who have pain?
7. The mindfulness group is designed to help not just with pain but with other things going on in your life. Keeping this in mind, over the past 8 weeks while you were in the group, what do you think led to the biggest amount of day-to-day stress in your lives—pain or other things? (stress, anxiety, anger etc.)	Was it mainly pain that was the hardest to deal with or was it stress/anxiety or other upsetting situations?
8. How has the group helped you to cope with your pain?	Is there any activity or exercise that helped you cope a bit better with your pain?
9. Did the group meet your expectations or was there something you would have hoped to get that you did not?	N/A
10. How did the structure of the group work for you—days/times?	Can you think of any other models for the group that would have been more convenient? e.g., daily group for one week during the summer? Longer sessions/ more sessions?
11. Do you have suggestions of things that could have been done to encourage your practice of mindfulness?	Do you think receiving texts or email alerts would be helpful?
12. Is there anything that could have been done to make you feel safer to talk/share your experiences?	N/A
13. Would you recommend the experience of being in the mindfulness group to another teen with pain?	If no, why not? If yes, why would you recommend it?
14. How do you think you will keep mindfulness in your life after the group?	N/A
15. Do you have any other comments that you would like to share with us about the group?	Positive/negative experiences? General thoughts and feelings about group?

**Table A2:** Select quotes illustrating main themes.

Theme	Sample Quotes
Mindfulness Skills	*“*[Mindfulness] *is like being in the moment. You have to pay attention to the things around you. I used to be like a zombie at school, I would just walk through the halls and blindly follow my schedule and I never paid attention to things, I am even doing better in school now”* (Participant, Fall group). *“We acknowledge that [the pain] is there but it doesn’t control me, like it kinda has been… I’ve learned ways to not push away but see it’s there and continue with my daily life”* (Participant, Fall group). *“I don’t think* [the mindfulness group] *really helped much with the physical pain, more just with the emotional like having to constantly worry about it or overthinking it or stressing out about it. It helped a lot with the other stuff that pain brings with it”* (Participant, Fall group).
Support	*“For the weather report, I was very conscious of what I said cause usually it was like a negative thing …so, I was trying to be conscious about it, but I was just, I was honest because I trusted everyone”* (Participant, Fall group). *“We didn’t feel bothersome by saying it all… because everyone could kinda relate to it”* (Participant, Spring group).
Group Exercises	*“This whole group was based around a lot of exercises and just having so many different exercises was a really, really good way for all of us to find our own individual one that worked for ourselves”* (Participant, Spring group). *“The whole-body meditation…I didn’t like…it brings my attention to the pain and I am usually the type of person who distracts myself so it didn’t work out for me”* (Participant, Spring group).
Empowerment	*“You are in a lot of pain and it was nice to try and just find a way to relax and when ice packs and heating won’t do. It is nice to try—it is a new aspect to look at”* (Participant, Spring group).
Program expectations	*“A lot of the time through the hospital … what I am hearing when I come here, it’s like surgeons checking me and telling what I already know. In the mindfulness group I was learning something new and not expecting anything from the people who are teaching us, I just… didn’t know what to expect so it is kind of exciting”* (Participant, Spring group). *“*[The mindfulness group] *is good to meet people, but don’t expect it to affect your physical pain, ‘cause I really had like my expectations of that”* (Participant, Fall group).
Logistics	*“I personally would like to have more sessions because 8 weeks is two months but now that we are at the end of two months it is just like … I don’t want to say goodbye”* (Participant, Spring group).
General Feedback	*“I think actually like everyone in this group, each of them have brought something new to mindfulness like I think we all make it our own sort of, and sometimes people will come in and share how they used it but in a different situation so it kinda helped me learn what other people are doing too and different perceptions and concepts - so it took away from having other people teach me too as well as the therapist but all my friends too”* (Participant, Fall group).
